# The reimplantation of anomalous aortic origin of the right coronary artery under lower mini-sternotomy

**DOI:** 10.1093/jscr/rjae528

**Published:** 2024-08-23

**Authors:** Koki Maekawa, Shota Yamanaka, Yohe Onga, Shu Takahashi, Taro Kanamori

**Affiliations:** Department of Cardiovascular Surgery, Kawaguchi Cardiovascular and Respiratory Hospital, 1-1-51, Maekawa, Kawaguchi, Saitama 333-0842, Japan; Department of Cardiovascular Surgery, Kawaguchi Cardiovascular and Respiratory Hospital, 1-1-51, Maekawa, Kawaguchi, Saitama 333-0842, Japan; Department of Cardiovascular Surgery, Kawaguchi Cardiovascular and Respiratory Hospital, 1-1-51, Maekawa, Kawaguchi, Saitama 333-0842, Japan; Department of Cardiovascular Surgery, Kawaguchi Cardiovascular and Respiratory Hospital, 1-1-51, Maekawa, Kawaguchi, Saitama 333-0842, Japan; Department of Cardiovascular Surgery, Kawaguchi Cardiovascular and Respiratory Hospital, 1-1-51, Maekawa, Kawaguchi, Saitama 333-0842, Japan

**Keywords:** anomalous aortic origin of the right coronary artery, lower mini-sternotomy, reimplantation

## Abstract

The patient was 28-year-old male. He was suffered from chest pain at rest. He was diagnosed with AAORCA (anomalous aortic origin of the right coronary artery) by emergency catheter. Myocardial scintigraphy indicated ischemic changes in the right coronary artery region, so surgery was the plan. Reimplantation was selected because the coronary artery computed tomography showed little intramural travel and mild coronary artery stenosis. The surgery was performed under lower mini-sternotomy to facilitate early return to work. The patient had a good postoperative course, and was discharged from the hospital postoperative Day 11 after rehabilitation. We report a case of the right coronary artery reimplantation with lower mini-sternotomy for AAORCA.

## Introduction

Anomalous aortic origin of a coronary artery (AAOCA) account for 1.3% of congenital heart diseases and are relatively rare [1]. Anomalous aortic origin of the right coronary artery (AAORCA) is more common than anomalous aortic origin of the left coronary artery (AAOLCA), but AAORCA often develops without symptoms and is often detected incidentally in adulthood by computed tomography (CT), CAG (coronary angiography), or other imaging studies. Treatment options include unroofing, reimplantation, and CABG (coronary artery bypass grafting), but the choice of procedure remains controversial. The reimplantation of AAORCA is usually performed through a median sternotomy to allow optimal access to the aortic root. In this case, we report on a reimplantation under lower mini-sternotomy.

## Case report

**Figure 1:**
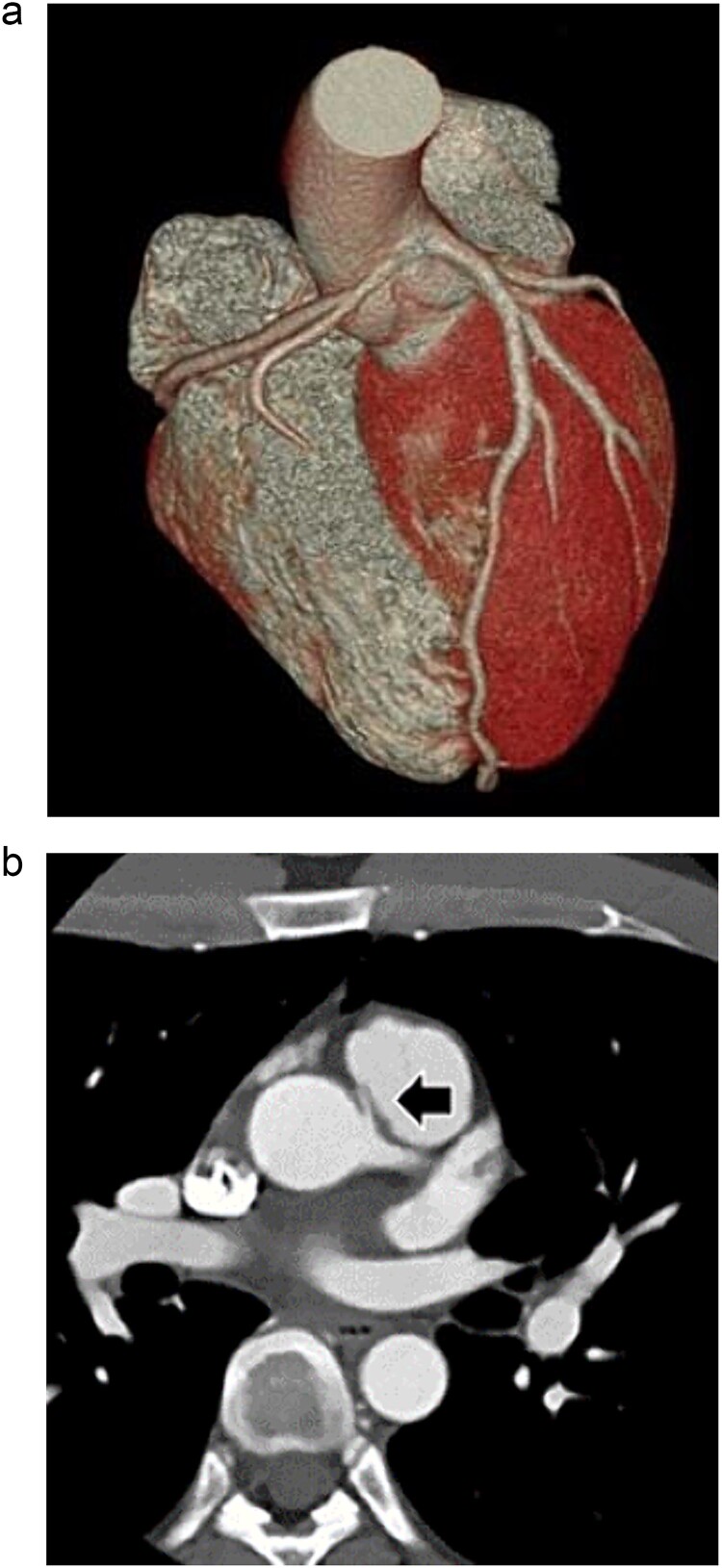
Preoperative three-dimensional CT, and enhanced CT shows AAORCA (a), and the right coronary artery (arrow) ran between the ascending aorta and the main pulmonary artery (b).

The patient was a 28-year-old male who had fainted during training a year earlier. He was brought to the emergency room with chest pain at rest. On arrival, his vitals were HR 82/min, regular, BP 116/65 mmHg, sPO^2^ 100% (3 L), and no ECG abnormality was noted. Echocardiography showed no evidence of decreased cardiac function, but blood test showed elevated myocardial troponin T 0.846 ng/ml. CAG showed no significant stenosis of the coronary arteries, and an abnormal right coronary artery origin was noted. Coronary artery CT showed that the right coronary artery ran between the ascending aorta and the main pulmonary artery. There was stenosis of the right coronary artery origin also noted (Fig. 1a, b). Furthermore, myocardial scintigraphy T1-201 I-123 BMIPP indicated ischemia in the right coronary artery region.

**Figure 2:**
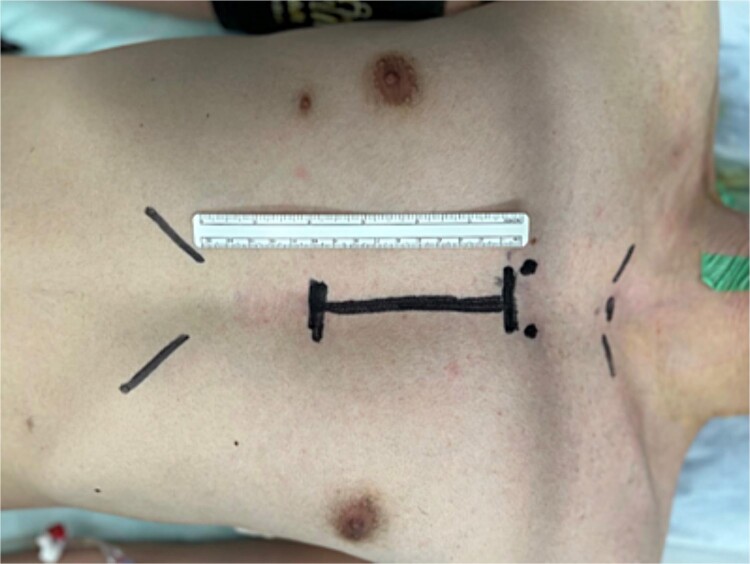
The surgery was performed under lower mini-sternotomy with a 10-cm skin incision.

**Figure 3:**
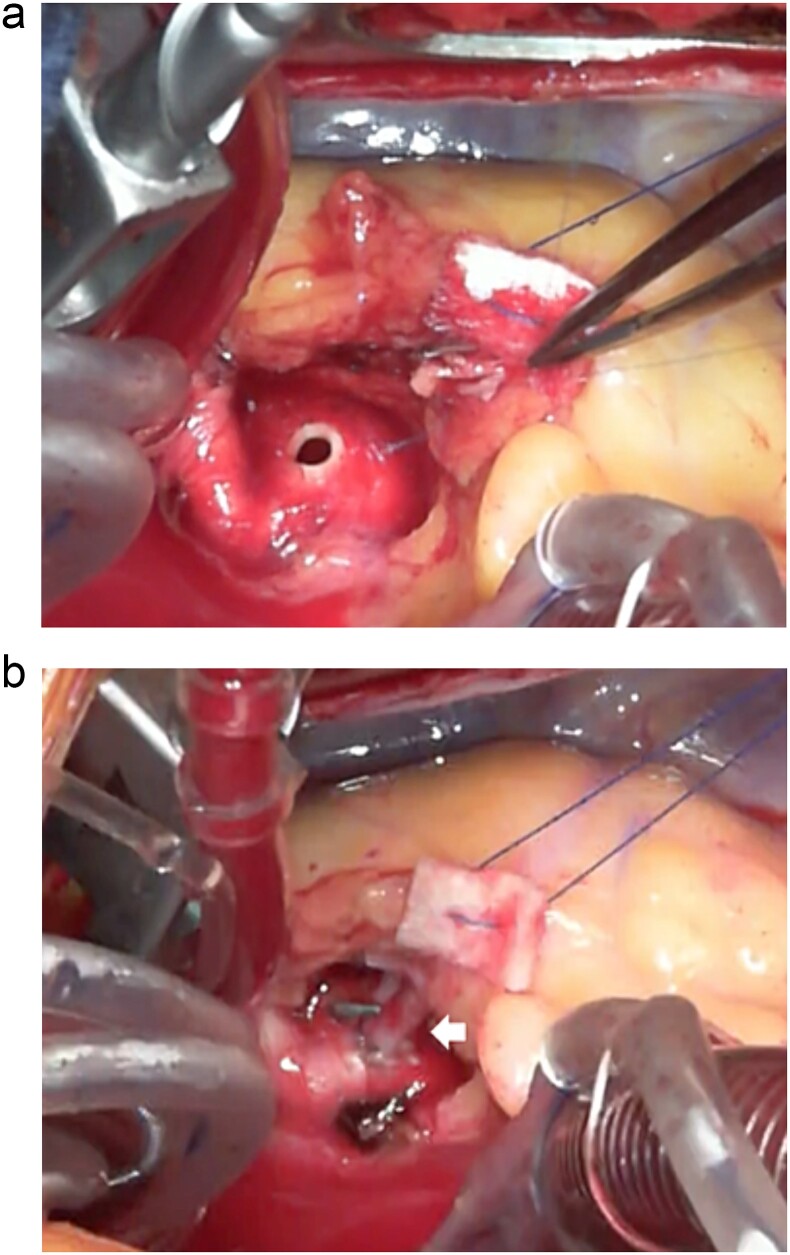
The hole was punched through the aorta with a 3.5-mm aortic punch (a) and the right coronary artery (arrow) was carefully fixed to the ascending aorta with 8-0 polypropylene continuous suture (b).

**Figure 4:**
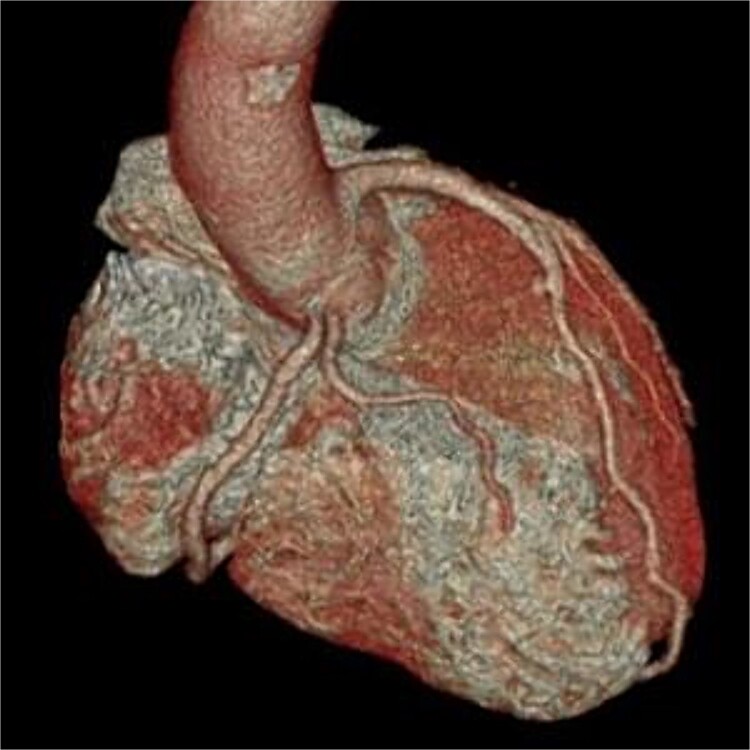
Postoperative three-dimensional CT, and enhanced CT shows the patency of the right coronary artery.

At surgery, a 10-cm skin incision was made. A J-shaped lower sternal partial median incision was made in the right second intercostal space (Fig. 2). CPB (Cardiopulmonary bypass) was established with ascending aortic-right atrium circuit. The right coronary artery was dissected after cross clamp just after the stenosis at the beginning of the right coronary artery with a clip. After confirming the position of the right coronary artery with attention to bending, a hole was punched through the aorta with a 3.5-mm aortic punch. The right coronary artery was carefully fixed to the ascending aorta whit 8–0 polypropylene continuous suture (Fig. 3a, b). Weaning from the CPB was easy; cardiac arrest time was 45 min, aortic cross clamp time was 64 min, and operative time was 141 min. Postoperative CT confirmed the patency of the right coronary artery, and he was discharged on postoperative Day 11 with no complications (Fig. 4). He returned to work 2 weeks after leaving the hospital. It has now been 1 year since the surgery and he has been symptom-free in his daily life.

## Discussion

AAOCA has been associated with sudden cardiac death (SCD), particularly in young athletes. One previous autopsy study reported that AAOCA was the second most common cause of SCD in young competitive athletes [2]. Although AAORCA are more frequent than AAOLCA, the risk of sudden death is higher in AAOLCA, so surgery is recommended even if the patient had no symptoms.

The ACC/AHA guidelines [3] recommend surgery for AAORCA, which symptomatic and myocardial ischemia have been noted. Surgical treatment is also recommended for patients with ventricular arrhythmias even if asymptomatic or without myocardial ischemia. In addition, Nagashima *et al.* reported that in AAORCA with sandwiched between the aorta and pulmonary artery, risk factors for sudden cardiac arrest (SCA) were age 40 years or younger, male, sports activity, and steep angle of right coronary artery [4].

The choice of surgical technique is based on anatomical characteristics. In cases of intramural arteries, unroofing is the most common choice. Carlos *et al.* reported the surgical results of unroofing versus reimplantation in AAOCA, and found that reimplantation is a good indication for cases in which the coronary artery passes under the commissure or is compressed by the main pulmonary artery, and good results were reported [5]. In cases where the left and right coronary arteries have separate openings, reimplantation is often performed by dissecting the coronary arteries and grafting them in a position that does not cause stenosis or bending. CABG is also performed as a surgical treatment, but there is some uncertainty regarding the long-term results of bypass grafting in patients with no coronary artery stenosis or ischemia. On the other hand, CABG is considered a good indication for patients with significant stenosis [6]. Law *et al.* reported good mid-term results of reimplantation for symptomatic AAORCA [7] and, Furukawa *et al.* reported good long-term results of reimplantation for AAORCA [8]. In this case, the left and right coronary arteries opened separately at the height of the STJ (sinotubular junction), but the right coronary artery has a small distance within the aortic wall, and compressed by the main pulmonary artery. Reimplantation was performed to avoid stenosis and bending compression by main pulmonary artery.

Lower mini-sternotomy has advantages such as improved stability of the upper sternum and earlier return to work [9]. Although upper partial sternotomy is often used for surgery of the ascending aorta and aortic arch, a T-shaped lower partial sternotomy starting from the second intercostal space provides a better view for surgery of the aortic root [10]. We performed a J-shaped lower mini-sternotomy in this case. All cannulations were performed from the operative field, and the operative field could be developed without limitation of surgical manipulation. The lower mini-sternotomy approach was considered to provide easier access to the aortic root and better field development. Lower mini-sternotomy was selected for AAORCA to perform reimplantation with a good surgical field of view, enabling an early return to work.
